# DRhoGEF2 Regulates Cellular Tension and Cell Pulsations in the *Amnioserosa* during *Drosophila* Dorsal Closure

**DOI:** 10.1371/journal.pone.0023964

**Published:** 2011-09-16

**Authors:** Dulce Azevedo, Marco Antunes, Soren Prag, Xiaoyan Ma, Udo Hacker, G. Wayne Brodland, M. Shane Hutson, Jerome Solon, Antonio Jacinto

**Affiliations:** 1 Instituto de Medicina Molecular, Faculdade de Medicina da Universidade de Lisboa, Lisboa, Portugal; 2 Vanderbilt Institute for Integrative Biosystems Research and Education, Department of Physics and Astronomy, Vanderbilt University, Nashville, Tennessee, United States of America; 3 Department of Experimental Medical Science, Lund Strategic Research Center for Stem Cell Biology and Cell Therapy, Lund University, Lund, Sweden; 4 Department of Civil and Environmental Engineering, University of Waterloo, Waterloo, Ontario, Canada; 5 CRG - Centre for Genomic Regulation, Barcelona, Spain; 6 Instituto Gulbenkian de Ciência, Oeiras, Portugal; Northwestern University Feinberg School of Medicine, United States of America

## Abstract

Coordination of apical constriction in epithelial sheets is a fundamental process during embryogenesis. Here, we show that DRhoGEF2 is a key regulator of apical pulsation and constriction of amnioserosal cells during *Drosophila* dorsal closure. Amnioserosal cells mutant for DRhoGEF2 exhibit a consistent decrease in amnioserosa pulsations whereas overexpression of DRhoGEF2 in this tissue leads to an increase in the contraction time of pulsations. We probed the physical properties of the amnioserosa to show that the average tension in *DRhoGEF2* mutant cells is lower than wild-type and that overexpression of DRhoGEF2 results in a tissue that is more solid-like than wild-type. We also observe that in the DRhoGEF2 overexpressing cells there is a dramatic increase of apical actomyosin coalescence that can contribute to the generation of more contractile forces, leading to amnioserosal cells with smaller apical surface than wild-type. Conversely, in DRhoGEF2 mutants, the apical actomyosin coalescence is impaired. These results identify DRhoGEF2 as an upstream regulator of the actomyosin contractile machinery that drives amnioserosa cells pulsations and apical constriction.

## Introduction

One of the most fascinating aspects of studying development is the opportunity of observing morphogenetic events in front of our eyes in real time. These morphogenetic events underlie shape changes and/or movements, mostly dependent on an intact actomyosin cytoskeleton (a network of actin filaments cross-linked with myosin II molecular motors). Actin filaments and myosin II generate tensile forces in individual cells that are transmitted across an entire tissue through adherens junctions (AJs) [1], [2]. During epithelial morphogenesis apical constriction is generated by this type of forces and results in a reduction of the cells' apical domain [3]. There are two main models to explain apical constriction. The first one, the purse-string model, proposes that stable contractile forces are generated by cortical myosin II driving sliding of actin filaments, while the second, the meshwork model, has been correlated with bursts of actin and myosin II, present in a medial zone, which generate more dynamic forces [4].

At the end of *Drosophila* embryogenesis, the dorsal region of the embryo is covered by a single layer of polygonal cells, named amnioserosa (AS). During dorsal closure AS cells constrict apically at the same time as the lateral epidermis moves to occupy their space. The tissue movements that characterise this complex morphogenetic event are driven by a combination of partially redundant forces [5], [6]. The first force to be identified is produced by actomyosin cables located at the leading edge of the dorsal-most epidermal cells, which have been proposed to function as a purse string that helps pulling the epidermis to the dorsal midline [7] through a ratchet-like mechanism [8]. As the epidermal sheets meet at the midline, the opposing leading edges zip up together to seal the epidermal discontinuity [9]. Concomitantly with these epidermal forces, the exposed AS surface area is actively reduced by the apical constriction of the AS cells [5], [10] due to forces that are produced both by cell–cell interfaces and by the cells' medial apical actin networks [11]. The mechanical coordination of tissue and cell behaviours is a crucial feature of dorsal closure that is particularly striking in the AS [12]. In spite of the global AS movement during dorsal closure being smooth each AS cell exhibits cycles of contraction and expansion, which are not synchronous but are coordinated in such a way that lead to continuous reduction of the AS dorsal surface [8]. A pulsating mechanism with similar mechanical properties seems to occur during gastrulation where the apical constriction of the ventral furrow cells is driven by pulsed contractions of an actomyosin network localised at the medial apical cortex [13]. Recently it has been shown that pulsed contractions in the AS are also associated with contractions of an apical actomyosin network and that those pulsations are regulated by the PAR complex [14] and by the Rho signalling pathway [15]. Expression of a constitutively active form of the myosin light chain kinase (ctMLCK) that increases myosin II activity, or expression of a constitutively active form of the formin Diaphanous (Dia^CA^) that stimulates actin polymerization, exhibited precocious cell contraction through changes in the subcellular localization of myosin II, demonstrating the role of these Rho1 effectors in the regulation of AS cell pulsations [16].

The upstream regulator of the Rho signalling pathway, RhoGEF2, was initially characterised as a regulator of apical constriction during formation of the ventral furrow [17], [18], [19] and has subsequently been shown to coordinate contractile forces throughout morphogenesis in *Drosophila* by regulating the association of myosin II with actin to form contractile cables [20]. Here, we show for the first time that DRhoGEF2 plays a crucial role in AS apical constriction through the regulation of myosin II subcellular localization and control of the AS cells pulsating behaviour upstream of Rho signalling.

## Results

### 1. DRhoGEF2 plays a role in Dorsal Closure

**Figure 1:**
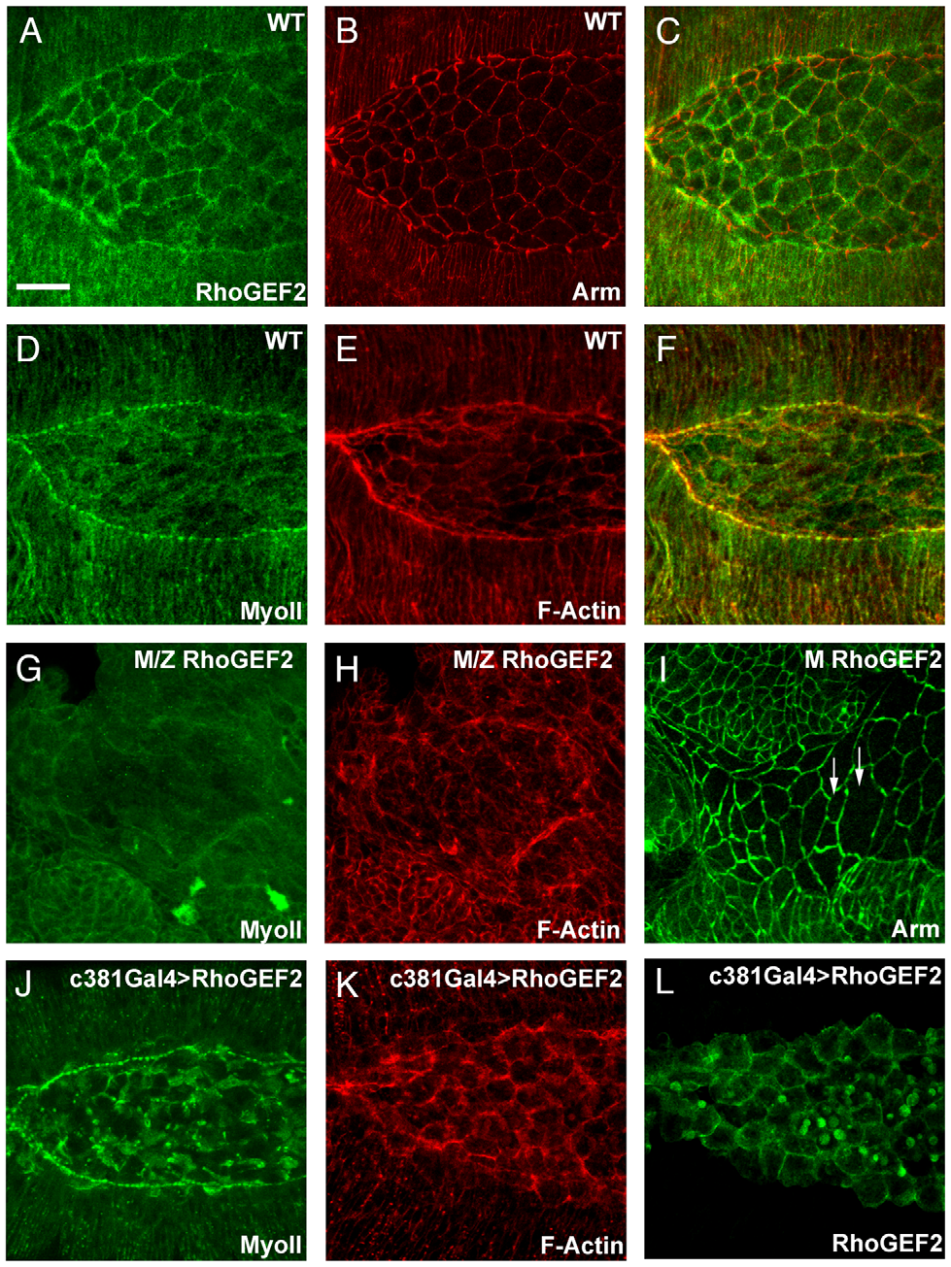
DRhoGEF2 plays a role in dorsal closure. (A) Anti-DRhoGEF2 staining showing that this protein is expressed in WT AS cells, the image shows only the most apical confocal sections of the AS cells. (B) Anti-Armadillo marks cell outlines of the cells at the level of the adherens junctions. (C) Merged image showing DRhoGEF2 and Armadillo. (D) Anti-myosin II staining marks the actomyosin cable and the AS cells. (E) Phalloidin staining marks actin filaments at the leading edge cable and cortical actin. (F) Merged image showing colocalisation of actin filaments and myosin II. (G) Myosin II staining in *DRhoGEF2* maternal zygotic mutants showing decreased levels of Myosin II. (H) Phalloidin staining showing that F-actin is also affected in *DRhoGEF2* zygotic maternal mutants. (I) Armadillo staining of *DRhoGEF2* maternal mutants exhibiting irregular actomyosin cables and abnormal AS cell shapes at different stages of dorsal closure. (J) Increased levels of MyoII in DRhoGEF2 overexpressing embryos. (K) Increased levels of F-actin in DRhoGEF2 overexpressing embryos. (L) Embryos overexpressing DRhoGEF2 specifically in AS cells stained for DRhoGEF2 to show the specificity of the driver. The scale bar represents 20 µm. During image acquisition we used the same parameters to allow the comparison of expression levels in different experiments.

DRhoGEF2 has been shown to be expressed in AS cells [20] but the analysis of the function of DRhoGEF2 during dorsal closure has been precluded by its earlier role during gastrulation. We started by confirming that DRhoGEF2 is indeed localized at the right place and time to play a role in dorsal closure. In wild-type (WT) embryos, DRhoGEF2 protein accumulates along the leading edge of the dorsal-most epidermal cells and apically in AS cells (Fig. 1A). DRhoGEF2 localization in AS cells is increased cortically (Fig. 1A–C, the outlines of the cells are marked by Armadillo).

To investigate whether DRhoGEF2 regulates apical constriction of AS cells during dorsal closure we took loss and gain of function approaches. *DRhoGEF2* maternal zygotic mutants showed significant changes of key components of the contractile machinery; myosin II was clearly reduced (Fig. 1G) and F-actin was more disorganised (Fig. 1H) in the AS cells when compared to WT (Fig. 1 D–F). However, as DRhoGEF2 plays an important role during gastrulation [17], [18], it was difficult to find embryos reaching dorsal closure stages, and the few that did were too abnormal for a more detailed analysis. To get around this limitation we used maternal mutants in which there is a paternal rescue allowing us to obtain embryos with reduced DRhoGEF2 function for analysing cell shape and dynamics. When stained for Arm to mark cell outlines (Fig. 1I), these *DRhoGEF2* maternal mutant embryos showed several tissue organization defects in the epithelial cells and in the AS. The leading edge of the dorsal-most epithelial mutant cells was irregular, in contrast to the WT (compare Fig. 1I with 1B). In the WT, all central AS cells showed similar exposed apical surface areas (Fig. 1B), whereas in the mutant, neighbouring AS cells presented very different apical areas (see arrows in Fig. 1I). In contrast to the mutant, overexpression of DRhoGEF2 in AS cells resulted in increased levels of myosin II and F-actin (compare Fig. 1J with 1D and Fig. 1K with 1E).

### 2. Cellular tension is affected in DRhoGEF2 mutants

**Figure 2:**
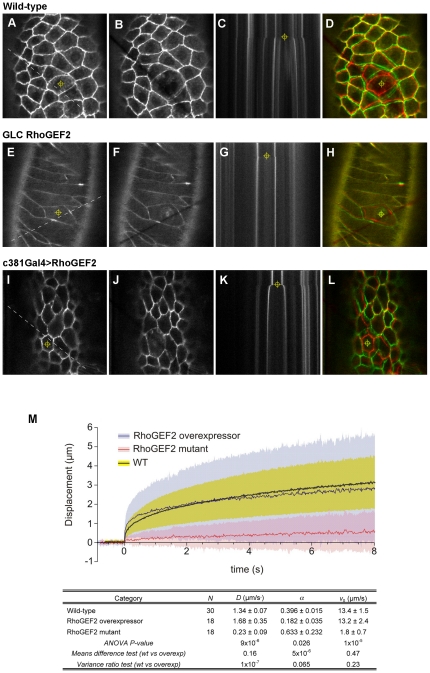
Cellular tension is affected when DRhoGEF2 expression levels are modified. (A–L) Amnioserosa mechanical response after ablation of a single cell. (A), (E) and (I) are confocal images from stage 13 embryos of wild-type, RhoGEF2 GLC and RhoGEF2 overexpression, respectively, before ablation. Dashed line represents the position of the line scanned repeatedly and used to build the kymographs (C, G and K). In (A), (E), (I), (D), (H) and (L) the crosshair indicates the ablated cell whereas in (C), (G) and (K) indicates the ablation time. (B), (F) and (J) are images from the same embryos taken after ablation of a single amnioserosa cell. (D), (H) and (L) are overlays of cell edges before (Red) and after (Green) ablation to illustrate each cell's recoil. (M) Mean recoil displacements for WT (blue), DRhoGEF2 overexpression (black) and DRhoGEF2 maternal mutants (red). The shaded areas represent the standard deviations. Displacement axis is in microns and time axis is in seconds. Note the higher SD in DRhoGEF2 overexpression (grey) compared with WT (yellow) and maternal mutants of DRhoGEF2 (pink). *v_0_* = initial recoil velocity; higher values indicate either more tension or less viscosity *D* = coefficient in power-law fit; higher values indicate either more tension or less stiffness *α* = power-law exponent; higher values indicate a more fluid tissue (lower values a more solid tissue) *v*_0_ was determined via a linear fit to the first 100 ms of recoil. *D* and *α* were determined via a power-law fit to the first 5 s of recoil.

In order to test whether DRhoGEF2 activity has a direct impact on tissue mechanics we assessed the cellular tension of the AS by performing a series of hole drilling experiments in embryos with reduced or increased DRhoGEF2 activity. We laser ablated a subcellular cylindrical hole through WT AS cells and we tracked the subsequent recoil of adjacent cells in order to calculate recoil parameters that allow us to evaluate cellular tension (see Fig. 2 (A–L) and Materials and Methods, [11]). The mean initial recoil velocity (*ν_0_*), determined via a linear fit to the first 100 ms of recoil, in the WT is 13.4±1.5 µm/s (Fig. 2M) whereas in the *DRhoGEF2* mutant it is 1.8±0.7 µm/s, which represents a decrease in the mutant of almost one order of magnitude, indicating that the mutant is under less tension and/or is more viscous. This result is in line with the value obtained for the coefficient *D*, calculated using a power-law fit to the first 5 s of recoil (Fig. 2M). The lower value obtained for the mean *D* in the mutant (0.23±0.09) is also an indication that the tissue is under less tension than the WT (1.34±0.07). The values of exponent α suggest that the mutant tissue may be more fluid than WT (0.633±0.232 *vs* 0.396±0.015).

The mean *D* and mean *v_0_* for WT and DRhoGEF2 overexpression is not significantly different (Fig. 2M, see also [11]), indicating that either the tension in DRhoGEF2 expressing cells is similar to WT or that an increase in tension is compensated by an increase in viscosity and stiffness. However, the variance of *D* is higher when overexpressing DRhoGEF2, consistent with a wider distribution of recoil displacements as shown in the respective graph (Fig. 2M, grey and yellow shadows). Interestingly the decrease in exponent α when DRhoGEF2 is overexpressed indicates a transition to a more solid-like tissue. Exponent α varies between 0 and 1 and lower values are characteristic of more solid materials [21]. Taken together, the results of the hole drilling experiments support the hypothesis that DRhoGEF2 regulates tissue tension in AS cells. In particular, the average tension in *DRhoGEF2* mutant cells seems to be lower than in WT, and the overexpression of *DRhoGEF2* results in a tissue that is less fluid and more solid-like.

### 3. DRhoGEF2 regulates AS pulsations

**Figure 3:**
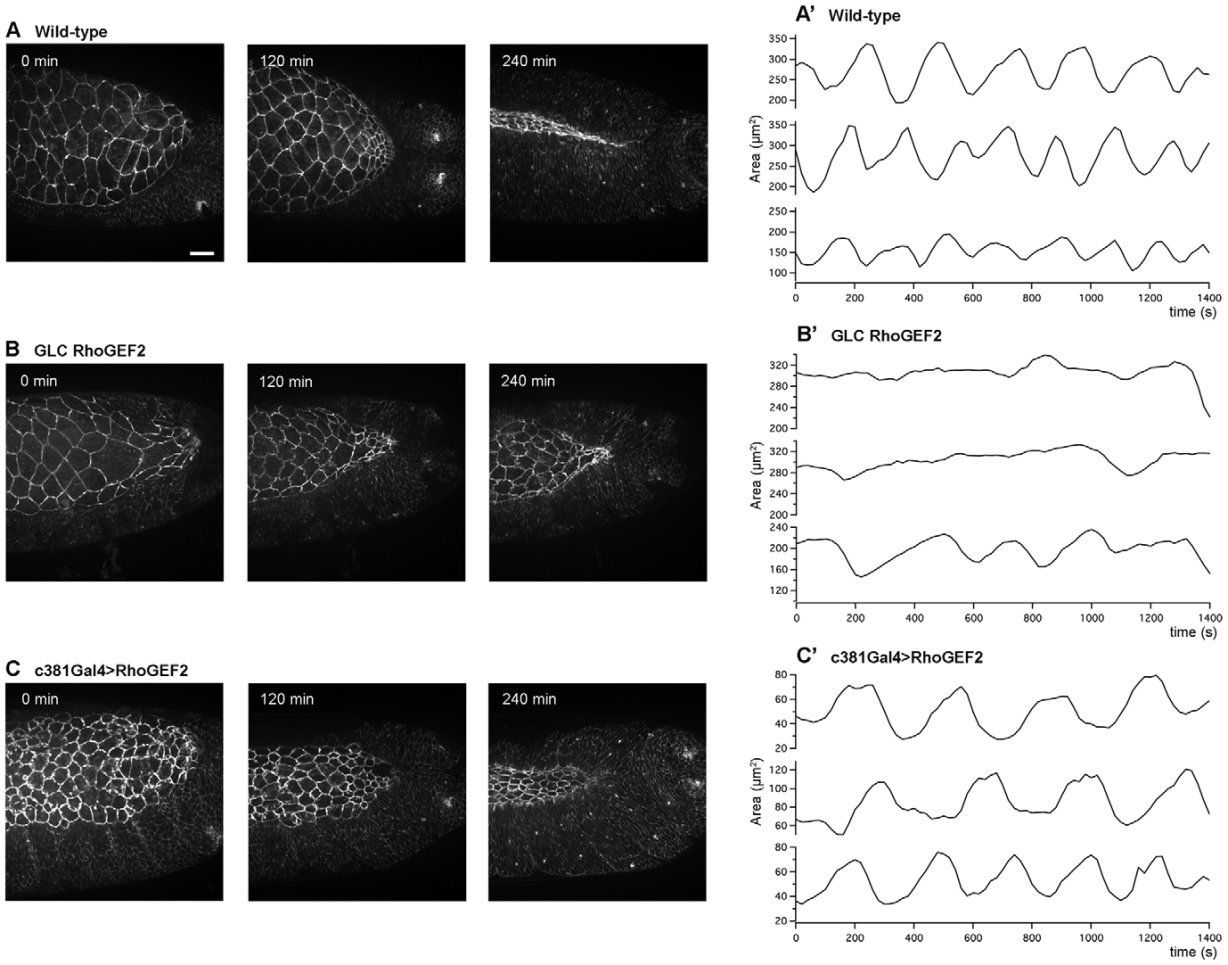
Loss and gain of function of DRhoGEF2 results in dorsal closure delay and impaired AS cell pulsations. (A–C) Stills from movies during dorsal closure in embryos marked with Ubi-DECadherin-GFP. (A) Wild-type. (B) Maternal *DRhoGEF2* mutants expressing Ubi-DECadherin-GFP. (C) Embryos marked with Ubi-DECadherin-GFP where UAS-DRhoGEF2 was overexpressed only in the AS cells. Embryos are shown at time 0, 120 and 240 min. Starting of dorsal closure (time 0) was considered when germ band was completely retracted. At 240 min, WT almost reach the end of dorsal closure, whereas *DRhoGEF2* maternal mutants and *c381GAL4/UAS-DRhoGEF2* holes are still open. Note that cell area is increased in *DRhoGEF2* maternal mutants and decreased in *c381GAL4/UAS-DRhoGEF2*. The scale bar represents 20 µm. (A′–C′) Apical cell surface area oscillations of three representative AS cells from (A′) Wild-type, (B′) *DRhoGEF2* maternal mutants, and (C′) *c381GAL4/UAS-DRhoGEF2*. Amplitude is in µm^2^ and time is in seconds (s). All AS cell pulsation analysis was performed on stage 13 embryos.

**Figure 4:**
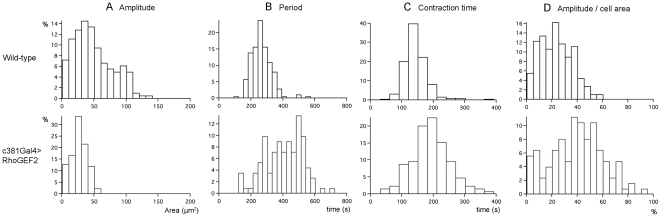
DRhoGEF2 plays a role in AS cell pulsations. Histograms of (A) Amplitude of area pulsations in µm^2^, (B) Period in seconds (s), (C) Contraction time in seconds (s), for wild-type and *c381GAL4/UAS-DRhoGEF2* AS cells and (D) Ratio of amplitude over cell area in percentage. In all histograms the X axis values have been normalized and presented as percentages. For WT the numbers of cells analysed was respectively 178, 172, and 178 for amplitude, period, and contraction, and for DRhoGEF2 overexpressing embryos the numbers where respectively 124, 111, and 124.

In order to find out whether DRhoGEF2 regulates AS pulsations, we investigated the dynamic behaviour of the AS cells in more detail by performing high speed time-lapse imaging with subcellular resolution (see Materials and Methods). The comparison of overall dorsal closure dynamics between WT and *DRhoGEF2* maternal zygotic mutants was not possible as the embryos with that genotype were extremely deformed. In *DRhoGEF2* maternal mutants, that were more amenable for time-lapse imaging, dorsal closure was slower than in WT but the phenotype was very variable (Fig. 3A–B). When DRhoGEF2 was overexpressed specifically in AS cells dorsal closure also took longer to be completed but, as described above, the average apical surface of the AS cells was significantly smaller than WT and the AS seemed more densely packed (Fig. 3C). To quantify the dynamics of dorsal closure in the different genotypes, we focused on early dorsal closure stages, starting at stage 13. In the WT (Fig. 3A′, Supplementary Movie S1), AS cells showed a cell pulsation period of 248±64 s, (Fig. 4B, upper graph) and an average cell area amplitude of 49±30 µm^2^ (Fig. 4A, upper graph), consistent with what has been previously described [8]. The analysis of *DRhoGEF2* maternal mutants revealed that the pulsation phenotype is variable, ranging from cells with almost no pulsations to cases that showed very irregular oscillations (see representative examples in Fig. 3B′ and Movie S2). In this case it was not possible to calculate a meaningful average period or amplitude, as the majority of the cells do not exhibit a clear periodic behaviour. Therefore, we conclude that DRhoGEF2 is required for AS cell pulsations.

In DRhoGEF2 overexpressing AS cells (Fig. 3C′, Movie S3) the amplitude of pulsations is decreased to 26±13 µm^2^ compared to 49±30 µm^2^ in WT (Fig. 4A), and period, 387±119 s, is longer when compared to 248±64 s in WT (Fig. 4B). For this genotype the distribution of amplitudes is clearly skewed towards lower amplitudes, however, the distribution of the ratios amplitude/cell area (Fig. 4D) shows that the relative amplitude is higher in DRhoGEF2 overexpressing AS cells. Interestingly, the time that these cells spend contracting (as opposed to expanding) also tends to be longer (Fig. 4C) indicating that enhanced DRhoGEF2 activity favours contraction. Supporting this possibility, we observed that at the end of germ band retraction, DRhoGEF2 overexpressing cells seemed to start constricting apically earlier than WT (Movies S4 and S6); when dorsal closure started, most AS DRhoGEF2 overexpressing cells had a reduced and uniform apical surface whereas WT cells appeared larger with a more irregular shape, although cell number is equal (179±20 in WT *vs* 178.3±17 in DRhoGEF2). Furthermore, the total AS tissue area at the end of germ band retraction, in the different experimental conditions, is consistent with the effects at the cellular level; embryos where DRhoGEF2 is overexpressed and *DRhoGEF2* mutants presented respectively smaller and larger AS areas than WT (Movies S4, S5 and S6).

### 4.DRhoGEF2 regulates actomyosin coalescence

**Figure 5:**
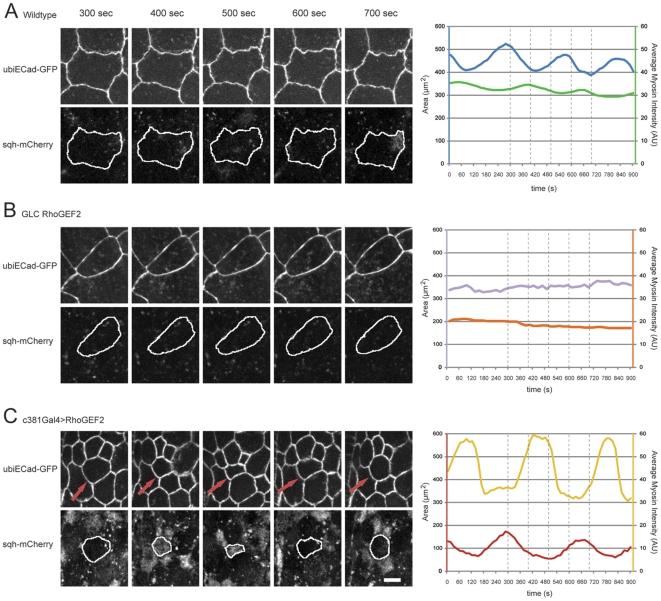
Pulsations in cell areas are asynchronous with fluctuations in actomyosin coalescence. Still images of a representative AS cell expressing UbiDECad-GFP and Sqh-mCherry captured from time series of (A) Wild-type, (B) *DRhoGEF2* maternal mutants, and (C) *c381GAL4/UAS-DRhoGEF2*, from stage 13 embryos. Cell areas were measured in the UbiDECad-GFP channel, and average intensity of myosin II was measured in Sqh-mCherry channel in the corresponding cell area. Quantified analyses from 15 second time intervals are presented. On each graph the lines corresponding to cell area and average myosin II intensity are colour coded according to the respective axis. The still images highlight maximum and minimum of cell area and myosin II intensity, and the time points are indicated in the graph by vertical dotted lines. Bar indicates 10 µm.

**Figure 6:**
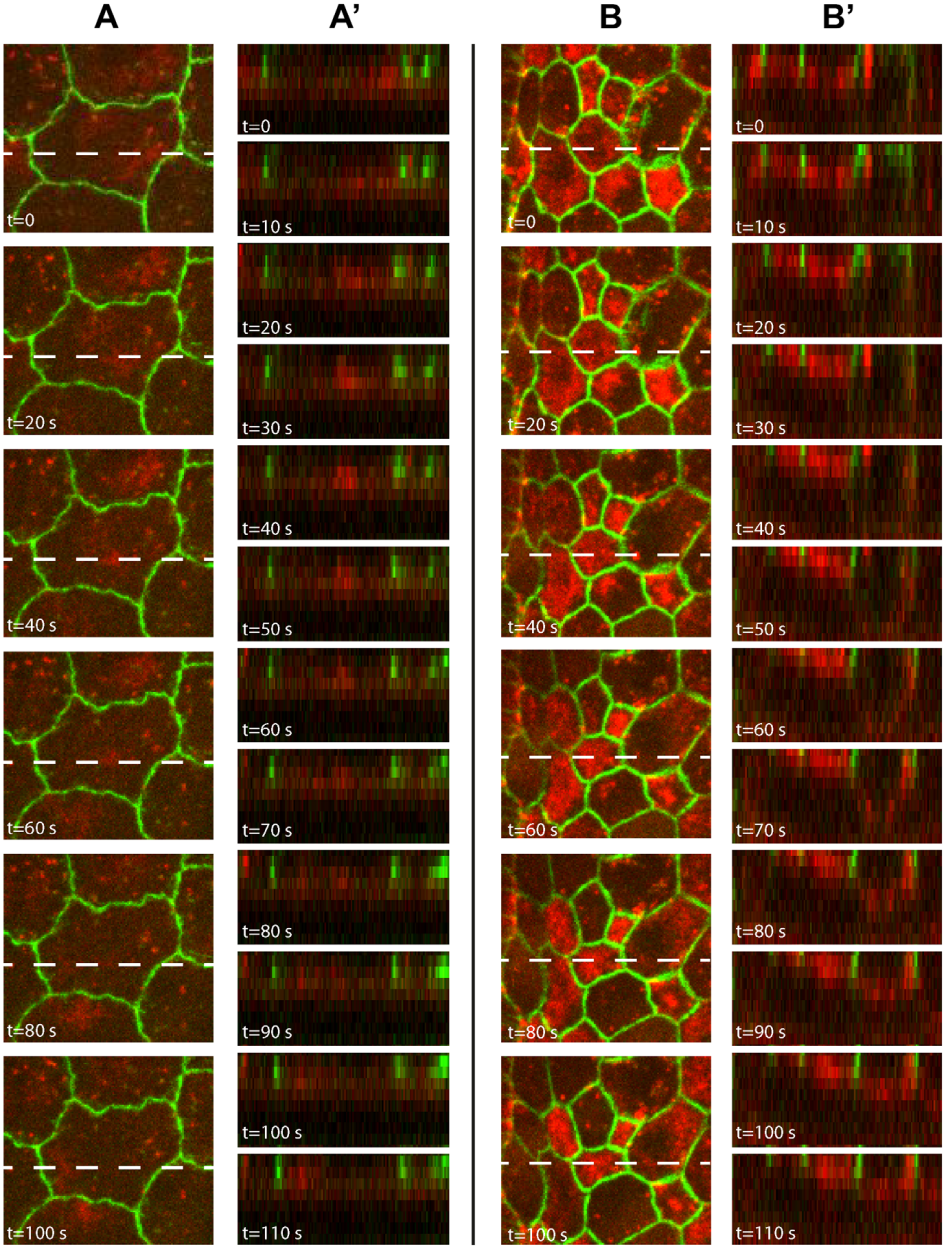
Distribution of Myosin II in WT and DRhoGEF2 overexpressing AS cells. Still images of a representative AS cell, expressing DE-Cadherin-GFP and Myosin mCherry, captured from time series at indicated time points of (A) Wild-type, (B) *c381GAL4/UAS-DRhoGEF2*, and Z-sections of (A′) Wild-type, and (B′) *c381GAL4/UAS-DRhoGEF2*. Dashed lines indicate plane for transverse sections.

The pulsating mechanism existing in the ventral furrow cells during gastrulation and recently also shown in AS cells, is dependent on an actomyosin network [13], [14], [16]. Thus, we investigated whether DRhoGEF2 regulates these cytoskeleton structures. Actin or myosin II mCherry-based probes were expressed simultaneously with DECad-GFP to examine the correlation between actomyosin coalescence and AS pulsation. As both actin and myosin II showed similar behaviour (results not shown, [16]) we hereafter refer to it as actomyosin. In WT AS cells we observed reciprocal fluctuations between AS cell area and actomyosin coalescence (Movie S7). When actomyosin coalescence was reduced, the area of AS cells was increased (Fig. 5A, time 300 s). Conversely, as actomyosin coalescence increased, we observed a subsequent decrease in AS cell area, and when actomyosin coalescence reached maximum intensity, the AS cell area was at its minimum size (Fig. 5A, time 400 s). In *DRhoGEF2* maternal mutants, the AS cells did not show a clear pulsating behaviour (Fig. 5B and Movie S8) and the lack of pulsations correlated with the absence of actomyosin coalescence (Fig. 5B). In contrast, in DRhoGEF2 overexpression, we observed reciprocal fluctuations between cell area and actomyosin coalescence, similarly to wild-type (Fig. 5C). However, actomyosin coalescence in WT AS cells fluctuated in a smoother manner, whereas in the DRhoGEF2 overexpression there was an extended lag period of low actomyosin levels and a sharp increase in coalescence (Fig. 5C, time 300 s and Movie S9). This shows that regardless of constitutive overexpression of DRhoGEF2, coalescence of actomyosin still fluctuates and contributes to generate AS cell pulsations. Interestingly, in WT cells the actomyosin coalescence appears locally in discrete areas of the AS cells, whereas in DRhoGEF2 overexpressing AS cells, it starts by distributing throughout the entire cell and becomes reduced to intense spots in the central region at the end of contraction. Consequently, we observed a discrete local contraction of the WT AS cells, and a more uniform contraction of the whole cell when overexpressing DRhoGEF2. To distinguish which myosin subpopulation, apical or junctional, is more affected when DRhoGEF2 is overexpressed, we analysed Z-stacks of pulsating AS cells. In both WT and overexpressing DRhoGEF2 AS cells, the mobile myosin fraction was located apically during the pulse, suggesting an association with a medial actomyosin meshwork (Fig. 6). However, upon overexpression of DRhoGEF2 in the AS cells, we observe higher levels of myosin contents correlated with a higher AS cell contraction (compare cell diameter in Fig. 6A and B) suggesting that DRhoGEF2 dependent pathways are activated. We do not observe any significant myosin localization at the adherent junctions where cadherin is localized.

The levels of apical myosin accumulation also correlate with the waviness of the membranes. In the WT the membranes are wigglier whereas in DRhoGEF2 they seem to be more isotropic (Fig. 6).

**Figure 7:**
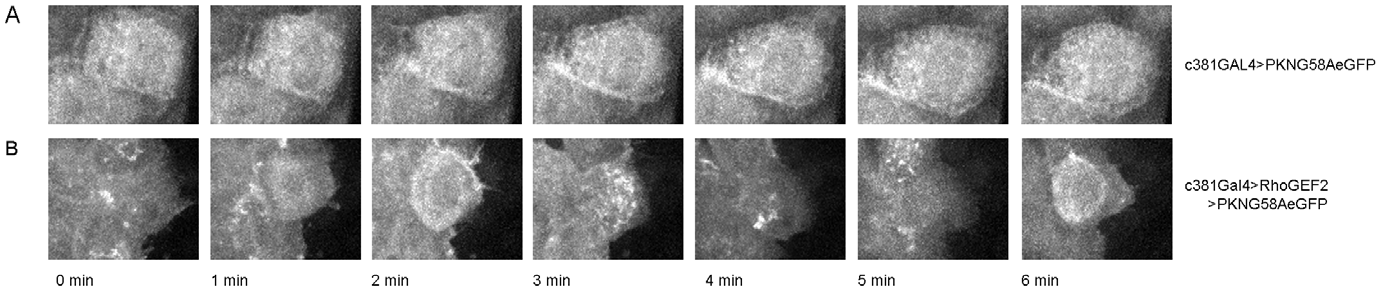
Rho1 is downstream of DRhoGEF2 regulating AS cell pulsations. Stills from movies during DC in embryos marked with the Rho1 probe PKNG58AeGFP. (A) *c381Gal4/UAS-PKNG58AeGFP* (B) *c381Gal4/UAS-DRhoGEF2;UAS-PKNG58AeGFP* (frames correspond to 19–25 mins from Movie S9). Note how Rho1 activity seems to be correlated with AS cells pulsation in terms of periodicity (see also Movie S9). The probe is not sensitive enough to clearly detect the local fluctuations of Rho1 activity in the WT AS cells.

To confirm that DRhoGEF2 is acting upstream of Rho1 activity in the AS cells we used a GFP based probe designed to detect GTP-bound Rho1 [22]. This probe is not sensitive enough to detect the local fluctuations of activity in the WT AS cells, but when we overexpress DRhoGEF2 we observe pulsations of Rho probe accumulation with a 4 minute period, which matches the apical pulsations (see Fig. 7 and Movie S10).

## Discussion

AS tissue mechanics plays a major role in dorsal closure [12]. The apical constriction of the AS cells results from asynchronous AS cell pulsations, followed by the contraction of an actomyosin purse string in the dorsal-most leading edge epidermal cells that acts in a ratchet-like manner [8]. In this study, we show that DRhoGEF2 controls AS pulsations through the regulation of periodic medial apical coalescence of actin and myosin II that flows across cell apices (our study and [16]). The normal pattern of AS pulsations is perturbed when the myosin II coalescence is altered by changing DRhoGEF2 expression. In particular, low DRhoGEF2 expression causes abolishment of the medial located myosin II coalescence, decreased pulsation amplitudes and aberrant pulsation periods. Conversely, increased DRhoGEF2 expression levels enhance myosin II coalescence thus reducing the cell's apical area and pulsation amplitude. Consequently the pulsation periods are extended, possibly due to the cells spending more time in a contracted state. In spite of the delay in dorsal closure caused by overexpression of DRhoGEF2 the ratchet mechanism does not seem to be significantly affected as in these embryos the actomyosin cable tightens up in the final phase of DC (See Movies S4 and S6).

The results of our hole drilling experiments are consistent with a role of DRhoGEF2 in the regulation of the forces that drive the pulsations. The results support the hypothesis that average tension in *DRhoGEF2* mutant AS cells is lower than WT, and that overexpression of DRhoGEF2 result in an AS tissue that is less fluid and more solid-like. When DRhoGEF2 is overexpressed there is more myosin II coalescence that can generate more contractile forces, leading to a more rigid tissue with smaller cells than WT. Surprisingly, our results suggest that the tension in DRhoGEF2 expressing cells is similar to WT. This may be explained by the existence of a tension plateau above which the forces generated and exerted by the cells lead to cellular and sub-cellular rearrangements that limit the amount of tension that we may observe, or alternatively, that an increase in tension is compensated by an increase in viscosity and stiffness. These results confirm previous studies showing that AS cellular tension is not only generated by cortical myosin II but also in at the medial region of the cell, where myosin II accumulates in discrete foci [11]. Together these data suggests that the medial actomyosin is a key factor for the generation of the forces that drive AS cell pulsations during early dorsal closure.

It is likely that Rho1, Diaphanous (Dia) and Myosin Light Chain Kinase (MLCK) are involved in myosin II localization and apical constriction downstream of DRhoGEF2. Indeed, expression of a dominant negative form of Rho1 blocks AS pulsations and expression of constitutively activated forms of its effectors Dia and MLCK, Dia^CA^ and ctMLCK respectively, results in the premature contraction of the AS and changes in the subcellular localization of myosin II [16]. The overexpression of Dia^CA^ and ctMLCK seem to maintain the contractile machinery in a constant overactivated state that leads to a reduction of AS apical surface, unusual cell shapes, and reduction of pulsations, or their complete arrest in the case of Dia^CA^ [16]. Interestingly, in our study, overexpression of DRhoGEF2 changes dramatically the dynamics of the pulsations and actomyosin coalescence without dampening them down significantly, the period is longer, the amplitude/cell area is higher, and the intensity of medial apical myosin II coalescence is considerably higher during the AS cells contraction phase. Our results show that activating the signalling cascade above the Rho1 switch, by overexpressing DRhoGEF2, does not lock the effectors in a permanent active state but leads to cycles of pulsations where the intensity of the contraction phase is higher than in WT.

A recent study has reported that cell polarity regulators Par-6, aPKC and Bazooka (Baz), are also involved in the control of AS pulsations during dorsal closure. The PAR complex accumulates at the apical surface of AS cells and regulates medial apical actomyosin coalescence; Baz seems to promote the duration of actomyosin pulses while Par-6/aPKC promotes the lull time between pulses [14]. It is possible that DRhoGEF2 constitutes a link between these upstream regulators and the Rho1 effectors, Dia and MLCK, which act directly on the actin filaments and myosin motors that generate force. It is also tempting to speculate that Rho kinase, a key mediator of Rho1 activity that can regulate Baz localization [23], may establish a feedback loop that regulates the cycles of pulsation.

Cell pulsations driven by the actomyosin network seem to be important features of several morphogenetic movements. The role of apical pulsations in dorsal closure and ventral furrow formation is now well established and some of the molecular players that regulate these processes have been identified. In this study we show for the first time that DRhoGEF2 is one of such players by controlling actomyosin coalescence and AS pulsations. The challenge for the future is to understand how the different players are connected to regulate these fascinating cyclic behaviours.

## Methods

### Fly stocks and genetics

UbiDECad-GFP and OR^R^ [24] were used as controls. The localisation of actin and myosin II was monitored using Sqh-mCherry [13] and sGMCA [5]. UAS lines were expressed using the UAS/GAL4 system [25]. UAS-DRhoGEF2 was described in [20]. GAL4 line c381 (expressed in the entire AS starting at stage 12) and *DRhoGEF2^I(2)04291^* were provided by the Bloomington Stock Centre. Germline clones of *DRhoGEF2^I(2)04291^* were generated using the FLP-DFS system [26], 48–72 hour larvae were heat shocked for 1 hour at 37°C. Rho1 activity was detected using the Rho1 sensor UAS-PKNG58AeGFP [22].

### Antibody stainings and fluorescent probes

Embryos were fixed according to [27]. The following antibodies were used: mouse anti-arm 1/50 (from Developmental Studies Hybridoma Bank), rabbit anti-DRhoGEF2 (1∶500; a kind gift from S. Rogers, DBCCGS, NC, USA), anti-myosin II (1∶500; a kind gift from D. P. Kiehart, DB, DU, NC, USA). All secondary antibodies were used at 1/200 dilution: Alexa 488, Alexa 568 and Alexa 633 (Molecular Probes) and Alexa 594-phalloidin was used at 1 µg/ml.

### Image acquisition

Embryos were selected at stage 12/13 and mounted as described [28]. Images of fixed tissues and time-lapse data were recorded using respectively a Zeiss META or an Andor Revolution confocal microscope. We recorded 3–5 embryos for each of the genotypes analysed. Unless otherwise specified, all images shown are projections of Z-sections. We used the same imaging settings for comparison of WT and mutant embryos at same developmental stages. When protein expression levels were compared, images were equally adjusted. For fluorescence quantification, images were acquired from live embryos using a spinning-disc confocal (Andor Revolution).

### Laser microsurgery

To assess cellular tension and mechanics in mutant embryos, we performed laser hole-drilling experiments as described previously [11], except that we measured the time at which ablation occurs and we estimated the initial recoil velocity via linear regression of the first 0.1 s of data. To assess the significance of differences in the recoil parameters for WT, *DRhoGEF2* mutant and DRhoGEF overexpressor, we used a single-factor ANOVA. Further analysis specifically compared WT type and DRhoGEF2 overexpressing fly embryos in terms of the mean parameter values (Student's t-test) and their variances (F-test). Regression and statistical analysis was performed in Mathematica (Wolfram Research, Champaign, IL).

### Image processing and analysis

Time-lapse images were analyzed with MATLAB-based analysis software. Similarly to [8], the image processing is realized in four successive steps: (1) A background removal to spatially homogenize the fluorescence intensity. (2) An adaptive threshold to over-segment the image. Small segmented objects (smaller than 10 pixels) are automatically removed. (3) A reconstruction with a watershed algorithm. (4) A correction by removing boundaries containing less than 70% of the pixels detected in step 2. The position of the boundaries, centre of mass, and area of individual cells are automatically detected and traced over time.

Automated determination of the amplitude and periodicity of contractions have been made in two steps: (1) Each individual cell area variation curve has been smoothed with a cubic spline function to remove all non relevant local extrema. (2) The coordinates of the remaining local extrema were automatically detected. The amplitude periodicity and contraction time (time that cells spend contracting) were therefore calculated from these positions. For WT the numbers of cells analysed was respectively 178, 172, and 178 for amplitude, period, and contraction, and for DRhoGEF2 overexpressing embryos the numbers where respectively 124, 111, and 124.

We used the ImageJ software to quantify myosin II intensity and apical surface of AS in double marked images. AS cell areas were measured from calibrated images by tracing the DECad-GFP channel using ImageJ tracing tool. The selected outline of the AS cell (green channel) was superimposed to the corresponding myosin II (red channel), and the myosin II coalescence was estimated as average fluorescent intensity of Sqh-mCherry within the selected area.

## Supporting Information

Movie S1 AS cell pulsations in the WT. A short movie of an *UbiCadh-GFP,c381Gal4* embryo imaged using time-lapse confocal microscopy showing an early stage of dorsal closure. Note how AS cells pulsate. The total elapsed time is 37 min and the frame rate is 30 s/frame.
(MOV)

Movie S2 AS cell pulsations in DRhoGEF2 maternal mutants. A short movie of an *UbiCadh-GFP/DRhoGEF2^I(2)04291^* embryo imaged using time-lapse confocal microscopy showing an early stage of dorsal closure. Note how AS cells pulsation is diminished compared to the WT. The total elapsed time is 37 min and the frame rate is 30 s/frame.
(MOV)

Movie S3 AS cell pulsations upon DRhoGEF2 overexpression. A short movie of an *UbiCadh-GFP,c381Gal4/UAS-DRhoGEF2* embryo imaged using time-lapse confocal microscopy showing an early stage of dorsal closure. Note how AS cells pulsate with a different behavior compared to the WT. The total elapsed time is 37 min and the frame rate is 30 s/frame.
(MOV)

Movie S4 Germ-band retraction in WT. Movie of an *UbiCadh-GFP,c381Gal4* embryo imaged using time-lapse confocal microscopy showing germ-band retraction and beginning of DC. The total elapsed time is 300 min and the frame rate is 10 min/frame.
(MOV)

Movie S5 Germ-band retraction in DRhoGEF2 maternal mutants. Movie of an *UbiCadh-GFP/DRhoGEF2^I(2)04291^* embryo imaged using time-lapse confocal microscopy showing germ-band retraction. Note that some AS cells are bigger than WT. The total elapsed time is 500 min and the frame rate is 10 min/frame.
(MOV)

Movie S6 Germ-band retraction in upon DRhoGEF2 overexpression. Movie of an *UbiCadh-GFP,c381Gal4/UAS-DRhoGEF2* embryo imaged using time-lapse confocal microscopy showing germ-band retraction. Note that AS cells acquire a rounder shape from the beginning of germ-band retraction. The total elapsed time is 500 min and the frame rate is 10 min/frame.
(MOV)

Movie S7 Myosin coalescence in WT. A short movie of an *UbiCadh-GFP/Sqh-mCherry,c381Gal4* embryo imaged using time-lapse confocal microscopy showing an early stage of dorsal closure. Note that Myosin II coalescence is correlated with cell deformations. The total elapsed time is 1250 sec and the frame rate is 5 s/frame.
(MOV)

Movie S8 Myosin coalescence in DRhoGEF2 maternal mutants. A short movie of an *UbiCadh-GFP,Sqh-mCherry/DRhoGEF2^I(2)04291^* embryo imaged using time-lapse confocal microscopy showing an early stage of dorsal closure. Note the absence of Myosin II coalescence. The total elapsed time is 800 sec and the frame rate is 5 s/frame.
(MOV)

Movie S9 Myosin coalescence upon DRhoGEF2 overexpression. A short movie of an *UbiCadh-GFP,UAS-DRhoGEF2/Sqh-mCherry,c381Gal4* embryo imaged using time-lapse confocal microscopy showing an early stage of dorsal closure. Note that Myosin II coalescence is more intense. The total elapsed time is 1185 sec and the frame rate is 5 s/frame.
(MOV)

Movie S10 Rho1 activity upon DRhoGEF2 overexpression. A short movie of an *c381Gal4/UAS-DRhoGEF2;UAS-PKNG58AeGFP* embryo imaged using time-lapse confocal microscopy showing an early stage of dorsal closure. Note that Rho1 activity is correlated with AS cells pulsation. The total elapsed time is 30 min and the frame rate is 30 s/frame.
(MOV)
